# Lesion Profiles and Tissue Pathology of *Clinostomum* Metacercariae in *Oreochromis niloticus* From Lake Tana and An Adjacent Aquaculture Pond, Bahir Dar, Ethiopia

**DOI:** 10.1002/vms3.71314

**Published:** 2026-11-05

**Authors:** Gebyaw Menge Getnet, Mersha Chanie Kebede, Assaye Desta Amare, Ayenalem Shibabaw Atenaf, Assaye Wollelie Fentie, Hailu Mazengia

**Affiliations:** ^1^ Department of Veterinary Science College of Agriculture and Natural Resources Debre Markos University Debre Markos Ethiopia; ^2^ Department of Veterinary Pathobiology College of Veterinary Medicine and Animal Sciences University of Gondar Gondar Ethiopia; ^3^ School of Veterinary Medicine Bahir Dar University Bahir Dar Ethiopia; ^4^ Department of Veterinary Medicine College of Agricultural Sciences Woldia University Woldia Ethiopia

**Keywords:** *Clinostomum*, cross‐sectional, Ethiopia, Lake Tana, metacercariae, *Oreochromis niloticus*

## Abstract

**Background:**

*Oreochromis niloticus* is the most commercially important and widely cultivated freshwater fish species in Ethiopia due to its rapid growth rate, adaptability to different environmental conditions, tolerance to handling stress and high nutritional value. However, infection with *Clinostomum* metacercariae (MC) is a major problem for both aquaculture and wild fish populations and poses a significant zoonotic risk to human health.

**Objectives:**

This study assessed the lesion profiles and tissue pathology associated with *Clinostomum* MC infection in 72 Nile tilapia fish collected from the southern Gulf of Lake Tana and adjacent ponds.

**Methods:**

Using purposive sampling techniques, the purposive sampling strategy was utilized specifically for pathological and comparative burden profiling rather than estimating true population prevalence. The study design was cross‐sectional and conducted from January to May 2026. The *Clinostomum* MC were identified on the basis of their morphological features at the genus level. Parasite burden and histopathological lesions in the muscle, gill and liver were assessed using descriptive statistics and an independent *t*‐test.

**Results:**

The muscle exhibited the highest proportion of encysted MC among sampled fish (83.3%), followed by the gills (50%) and liver (36.1%). The total cyst burden was higher in fish collected from Lake Tana (239 cysts) than in the adjacent pond (57 cysts). Several gross and histological lesions were identified in the examined fish organs. Lamellar fusion (36.1%) and lamellar hyperplasia (33.3%) were the most common gill lesions, whereas the major muscle lesions observed were muscle fibre degeneration and disruption (66.6%) and cellular infiltration (33.3%). Sinusoidal dilation and congestion (13.88%) and hepatocyte vacuolation (11.1%) were the main microscopic lesions of the liver. The mean severity indices of gill, muscle and liver were greater in fish collected from Lake Tana compared to ponds. Among the examined organs, muscle was the most affected, followed by the gills, whereas the liver was the least affected. There were strong positive correlations between parasite burden and lesion severity indices in all examined organs.

**Conclusion and Recommendations:**

The results indicate that *Clinostomum* MC cause significant tissue injury in Nile tilapia. Strengthening aquaculture farm biosecurity, snail control, regular surveillance, proper fish cooking practices and additional studies using molecular techniques and assessing seasonal variation are recommended to reduce the effects of the parasite on fish and its public health risks.

## Graphical abstract

A cross‐sectional study assessed *Clinostomum* metacercariae–associated lesions in 72 Nile tilapia from Lake Tana and adjacent aquaculture ponds. Cysts were predominantly found in muscle, followed by gills and liver. Histopathology revealed inflammation, lamellar fusion, hyperplasia, congestion and parasitic cysts, with significantly greater lesion severity in Lake Tana fish.

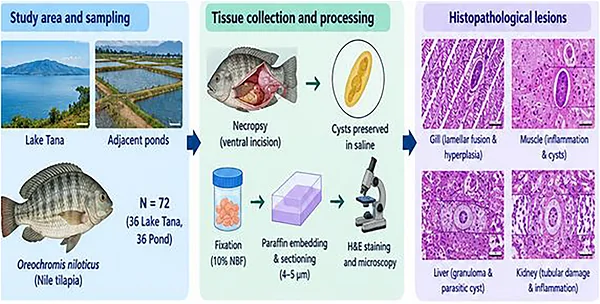

## 1 Introduction

Fish are the most diverse groups of vertebrates inhabiting a variety of marine and freshwater environments (Gulelat et al. 2013). It is one of the most important foods in the human diet because of its high nutritional content and is valued for improving diet quality (Tesfaye et al. 2023). Fish meat contains numerous beneficial components, including polyunsaturated fatty acids (PUFAs), essential minerals, omega‐3 and omega‐6 fatty acids, antioxidants, lipid‐soluble vitamins and easily digestible proteins, all of which play vital roles in promoting human health (Abd‐ELrahman et al. 2023; Utri‐Khodadady and Głąbska 2023).

Fisheries in Ethiopia exclusively depend on inland water bodies like lakes, reservoirs and rivers for fish supply and commercial production as a low‐cost protein source (Beletew et al. 2016). Recently, the government has given emphasis on fisheries and aquaculture as an important sub‐sector with potential to contribute to food security, employment creation and poverty reduction (Abiyu et al. 2020). The commercially most valuable fish species in Ethiopia include the *Oreochromis niloticus* and several species of the genera *Clarias*, *Lates*, *Barbus* and *Bagrus* (Gulelat et al. 2013)*. O. niloticus* is among the most widely cultivated freshwater fish due to its rapid growth rate, adaptability to a range of environmental conditions, resistance to handling stress and high nutritional value (Abd‐ELrahman et al. 2023).

Lake Tana represents one of Ethiopia's most important fishing grounds and is predominantly exploited by traditional fishermen (Beletew et al. 2016; Destaw et al. 2025). The lake supports a diverse community of fish species and harbours several unique endemic species, including the endemic subspecies of *O. niloticus tana* (Tefera et al. 2019). Moreover, Lake Tana sustains one of Ethiopia's most important inland fisheries, with *Labeobarbus* spp., African catfish (*Clarias gariepinus*) and Nile tilapia (*O. niloticus*) serving as the principal commercial gillnet fish species. These species contribute substantially to the total annual fish catch and play a vital role in local livelihoods and food security (Abiyu et al. 2020).

Similar to other vertebrates, fish play a significant role as the final or intermediate hosts for many parasites, with a potential for the zoonotic transmission of parasites to humans (Gulelat et al. 2013). Therefore, fish parasitic diseases are one of the potential factors that restrict the development of total fish production due to mortality, tissue damage, reducing their size, altering the behaviour of infected fish and making them vulnerable to other infections, resulting in higher mortality (Gulelat et al. 2013; Abd‐ELrahman et al. 2023; Yassen et al. 2023).

Among these, clinostomiasis is one of the parasitic diseases infecting freshwater fish caused by digenetic trematodes belonging to the family Clinostomatidae (Mai et al. 2021). This disease is a significant global concern because of its adverse effects on aquaculture productivity and potential risks to public health, because some *Clinostomum* metacercariae (MC) species may cause laryngo‐pharyngitis and Halzoun‐syndrome‐like disease in humans who eat improperly cooked food (Mai et al. 2021; Tesfaye et al. 2023). The *Clinostomum* MC, commonly referred to as yellow grubs, is marked with a bulge that contains a whitish‐yellow and elliptical or oval‐shaped body and can be observed on the skin, gills, muscle, or internal organs of fish and amphibians (Tesfaye et al. 2023).

The MC encyst within the tissues of the Nile tilapia fish, causing visible lesions and pathological changes that compromise fish health (Aydogdu et al. 2025). Infection with *Clinostomum* MC induces significant tissue alterations characterized by inflammatory reactions in both adipose and muscle tissues, necrosis, fibrosis, macrophage infiltration and degenerative changes in muscle fibre architecture (Mahdy et al. 2023). Moreover, the gill tissues exhibited marked pathological changes, including severe vascular congestion, haemorrhage, dense infiltration of inflammatory cells and extensive degeneration and loss of the lamellar epithelium (Mahdy et al. 2023; Yassen et al. 2023). The severity and distribution of these lesions depend largely on the parasite load and the host immune response (Gjurčević et al. 2022; Younis et al. 2026). Despite the importance of clinostomiasis in *O. niloticus*, there is limited information on the gross and histopathological lesions of *Clinostomum* MC in Lake Tana. This study aimed to investigate the lesion profiles and tissue pathology associated with *Clinostomum* MC infection in *O. niloticus* collected from Lake Tana and adjacent aquaculture ponds in Bahir Dar, Ethiopia, with emphasis on the pathological changes induced in different fish tissues and organs.

## 2 Materials and Methods

### 2.1 Study Area

The study was conducted on Lake Tana and adjacent ponds located in the northwestern highlands of Ethiopia, within the Amhara Regional State near Bahir Dar City. Lake Tana, the largest freshwater lake in Ethiopia, covers a surface area of approximately 3050 km^2^ (Beletew et al. 2016). Lake Tana is a shallow, turbid, and well‐mixed highland lake, with a mean depth of about 9 m (Assefa et al. 2019). The lake is situated at an altitude of 1830 m above sea level and serves as the source of the Blue Nile River. Geographically, it lies between 10°57′ and 12°47′ N latitude and 36°38′ and 38°14′ E longitude (Destaw et al. 2025).

#### 2.1.1 Description of Sampling Site

##### 2.1.1.1 Southern Gulf of Lake Tana

The southern gulf of Lake Tana, located near Bahir Dar, was purposively selected for this study due to ecological conditions favourable for the life cycle of *Clinostomum* MC. This area is frequented by various waterbirds, which serve as definitive hosts for the parasite, and contains extensive wetland habitats that support intermediate snail hosts (Figure 1) (Assefa et al. 2019).

**FIGURE 1:**
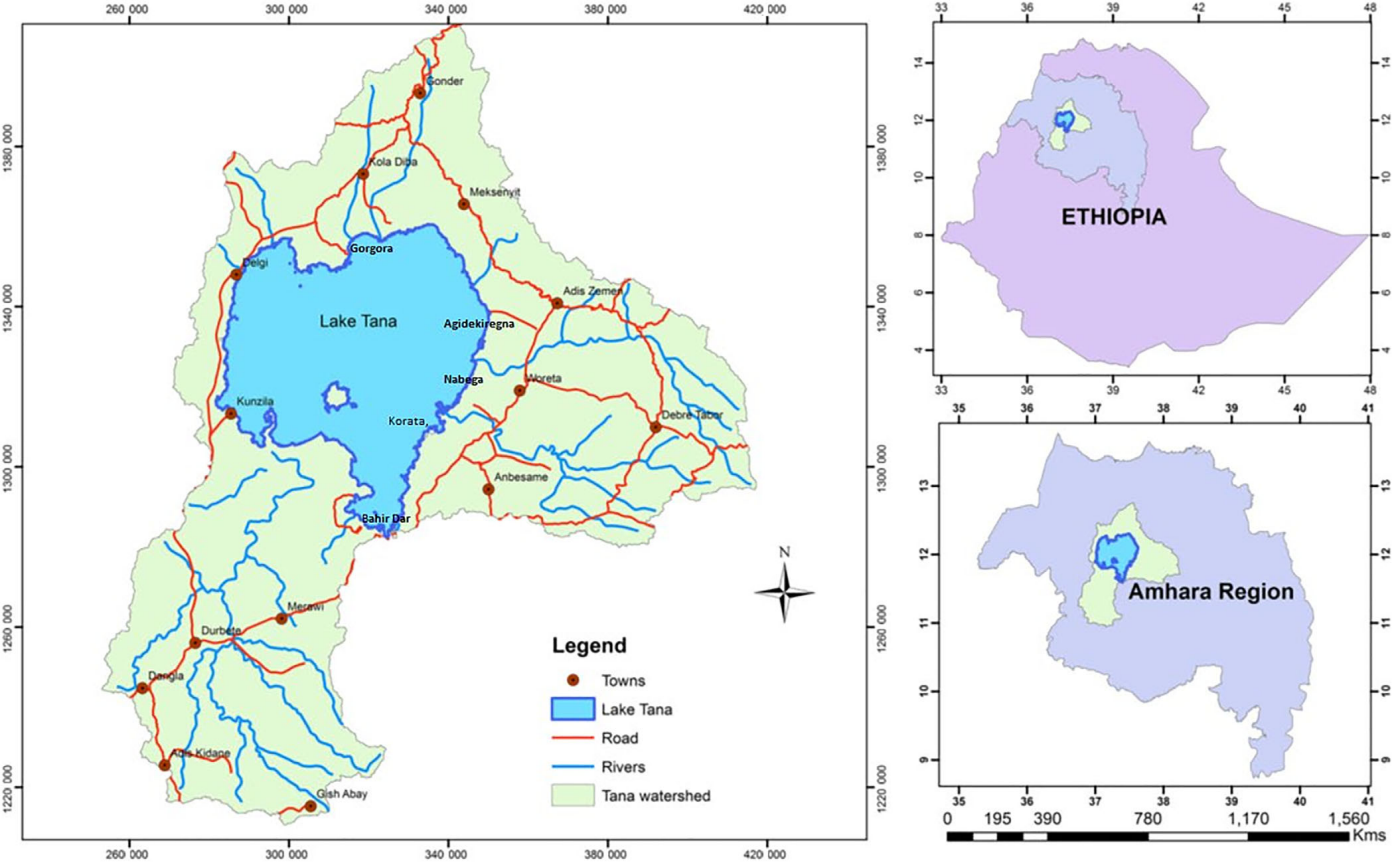
Map of study area and sampling sites (Assefa et al. 2019).

Human activities, including small‐scale aquaculture, fish markets and fishing practices, may further enhance interactions between fish, birds and snails, facilitating parasite transmission. Observations at this site indicate that the abundance of Nile tilapia is reduced compared to previous records (Getnet et al. 2024), likely influenced by ecological factors and increased exposure to parasite hosts.

##### 2.1.1.2 Nile Tilapia Fish Aquaculture Farm

The Nile tilapia farm is located at the Bahir Dar City Fisheries and Aquatic Research Center, adjacent to the southern gulf of Lake Tana. The farm is a separate unit with regular water replacement. Fish are fed properly, and the ponds are fully cleaned once a year. There is no direct drainage of water from the farm. The area is fenced, and only Nile tilapia are raised using batch culture. However, no chemical or medicinal treatments are used in the ponds, and no extra fish health measures are taken (Getnet et al. 2024).

### 2.2 Study Population

The study population was fish. The fish species selected was Nile tilapia (*O. niloticus*), locally known as ‘koroso’, because it is one of the most commercially important fish species in Lake Tana and plays a significant role in the local fishery and food supply (Abd‐ELrahman et al. 2023). Specimens were purchased directly from local fishermen operating in Lake Tana and adjacent ponds.

### 2.3 Study Design

A cross‐sectional study design was conducted from January to May 2026 to investigate the lesion profiles and tissue pathology associated with *Clinostomum* MC infection in *O. niloticus* from Lake Tana and adjacent ponds in Bahir Dar, Ethiopia. The study design integrated field parasitological studies with laboratory‐based gross and histopathological analyses, enabling both qualitative and quantitative characterization of tissue lesions.

### 2.4 Sample Size Determination and Sampling Techniques

The purposive sampling technique was employed by selectively obtaining fish infected with *Clinostomum* MC. A total of 72 *O. niloticus* (36 from each site) were examined. The sample size was determined primarily by the feasibility and availability of specimens during the study period and was considered appropriate for the exploratory and purposive nature of the study. The sample size was also guided by the number of specimens used in similar studies. As this was an exploratory study based on purposive sampling, a formal statistical power calculation was not performed. Fish were identified as infected on the basis of the presence of visible cysts on the gills, or buccal cavity, intramuscular and livers through superficial incision of tissues. Additionally, fish showing discolouration, excessive mucus secretion or tissue injury associated with the cysts were included (Mai et al. 2021; Yassen et al. 2023).

Previous studies on African freshwater fish health and parasitology have commonly employed sample sizes of 60 fish or more to achieve reliable statistical inference (Marancik et al. 2020; Akinjogunla and Adefiranye 2025). Moreover, large‐scale parasitological surveys examining over 400 fish, including approximately 72 individuals per species, have been documented (Amare et al. 2014). Therefore, a total sample of 72 Nile tilapia was consistent with prior studies.

### 2.5 Sample Collection and Transportation

Fish sample collection at each sampling site was carried out with the assistance of professional fishermen using seine nets and fishing boats. Immediately after capture, live fish were placed in aerated plastic containers containing native lake or pond water to minimize handling stress (Mahdy et al. 2023). Fish specimens were transported to the laboratory of the Bahir Dar Fisheries and Aquatic Research Center for detailed examination. Upon arrival to the laboratory, gross lesion observations were conducted, and the representative gill, muscle and liver organ samples were collected. Subsequently, the collected samples were transported to the Veterinary Pathology and Clinical Pathology Laboratory for histopathological examination. Each specimen was assigned a unique identification code, and the following data were recorded: sampling site (lake or pond) and date and time of capture.

### 2.6 Morphological Identification of Parasites

Observed cysts from fish organs were gently excised using fine forceps and washed with 0.65% saline and preserved in 70% ethanol and 5% glycerine for counting and morphological characterization (Mai et al. 2021). The recovered MC were examined using the naked eye, a stereomicroscope and an Olympus CX21/55 binocular light microscope to document morphological features, including body outline, sucker arrangement, reproductive organ arrangement and cyst wall structure. Identification was conducted on the basis of the morphological identification criteria described by Choudhary et al. (2022).

### 2.7 Assessment of Parasitic Burden

The parasitic burden of *Clinostomum* MC was measured for each fish and for each individual fish organ. First, the total number of cysts in the entire fish was counted. Subsequently, the number of cysts in each organ, including the gills, muscles and livers was recorded. For each fish, the proportion of cysts in each organ relative to the total number of cysts was calculated and expressed as a percentage frequency. This approach provided a detailed assessment of parasite load and demonstrated the distribution of parasites across different organs (Yassen et al. 2023).

### 2.8 Gross Examination

During the examination, each Nile tilapia fish was first subjected to a thorough external inspection to identify visible pathological changes and detect visible cysts. Particular attention was given to the gills, oral cavity, liver, operculum and musculature for the presence of pathological changes and encysted MC (EMC). These observations were recorded to detect external appearances associated with parasitic infection.

Following the external examination, a systematic necropsy was performed. A ventral midline incision was made extending from the anus to the operculum in order to expose the visceral organs. The internal organs, including the musculature, liver, spleen, kidney and gastrointestinal tract, were carefully examined for the presence of EMC (Mai et al. 2021). Due to the unavailability of chemical anaesthetics during the study period, fish were euthanized using cervical dislocation. This method involved the rapid severing of the spinal cord at the junction between the skull and the first cervical vertebra, ensuring immediate loss of neural function (Destaw et al. 2025).

### 2.9 Histopathological Tissue Processing and Examination

Representative tissue samples (∼1 cm^3^) from both infected and adjacent non‐infected areas of muscle, gill and liver were collected. The tissues were fixed in 10% neutral buffered formalin for 24–48 h (Mahdy et al. 2024). Formalin‐fixed tissues were trimmed to 2–3 mm, dehydrated, cleared and embedded in paraffin wax. Tissue blocks were prepared, and then the tissues were sectioned at a thickness of 4–5 µm using a rotary microtome. Tissue sections were mounted on frosted‐end clean glass slides and stained with hematoxylin and eosin (H&E) according to standard histopathological procedures previously described by Aydogdu et al. (2025).

Histopathological examination was conducted using an Olympus CX21/55 binocular light microscope under 10× and 40× objective lenses to evaluate the nature, distribution and severity of tissue alterations, following the method of Yassen et al. (2023). Lesions were categorized according to the type of tissue injury, including necrosis, fibrosis, haemorrhage and degeneration, as well as by the localization of metacercarial cysts within the affected tissues. In addition, lesions were classified on the basis of the nature of the inflammatory response, including granulomatous reaction, cellular infiltration and melanomacrophage aggregation centres. Furthermore, lesions were evaluated according to the degree of organ damage and architectural disruption. The representative lesions were photographed using a digital photomicroscope for permanent documentation.

### 2.10 Lesion Severity Scoring in Nile Tilapia

Microscopical lesion severity in *O. niloticus* infected with *Clinostomum* MC was evaluated using a modified semi‐quantitative scoring system (0–3). The scoring method was adapted from Bernet et al. (1999). Lesions were assessed separately for muscle, gills and liver using organ‐specific criteria. Mean lesion severity and the correlation between parasite burden and lesion severity scores for each organ index and fish index were calculated per site, allowing comparison between the two sampling sites. Organ lesion index was calculated by multiplying the value of the importance factor (*w*) with the score value (*a*), and then summing all detected histopathological lesions in each organ. The fish index was calculated by the sum of all organ indices from an individual fish, including the gills, liver and muscles. These indices indicate the extent of histological alterations in the tissues by converting observed qualitative histopathological changes into quantitative values (Getnet et al. 2024).

### 2.11 Data Management and Statistical Analysis

All collected data, encompassing parasite counts and lesion scores, were entered into Microsoft Excel and analysed using Stata v.17. Descriptive statistics (frequency and percentage) were used to summarize the parasite burden and lesion severity in different sampling sites. Data normality was assessed using the Shapiro–Wilk test, and the data were approximately normally distributed. Inferential analyses included the following: An independent *t*‐test to compare lesion severity scores across organs associated with study sites and Pearson correlation analysis to assess associations between parasite load and lesion severity. A *p* value <0.05 was considered statistically significant. Results were presented through tables and photomicrographs.

### 2.12 Quality Assurance and Control

To ensure methodological accuracy and reproducibility, all dissection instruments and glassware were sterilized before use. All histological reagents were prepared freshly. Data entries were randomly re‐checked to detect transcription or scoring errors.

## 3 Results

### 3.1 Morphological Identification of Parasites

EMC recovered from different fish body parts examined macroscopically, as well as under a stereomicroscope and light microscope (Figure 2). Taxonomic identification was limited to the genus level because the morphological features observed were insufficient for species‐level differentiation. Morphologically identified by yellowish‐white to orange pea‐like cyst, stout, tongue‐shaped bodies, flat and non‐segmented structures with smooth lateral margins, absence of spines and the arrangement of sucker and reproductive organs, following the procedures described by Montes et al. (2020), Tesfaye et al. (2023) and Yassen et al. (2023).

**FIGURE 2:**
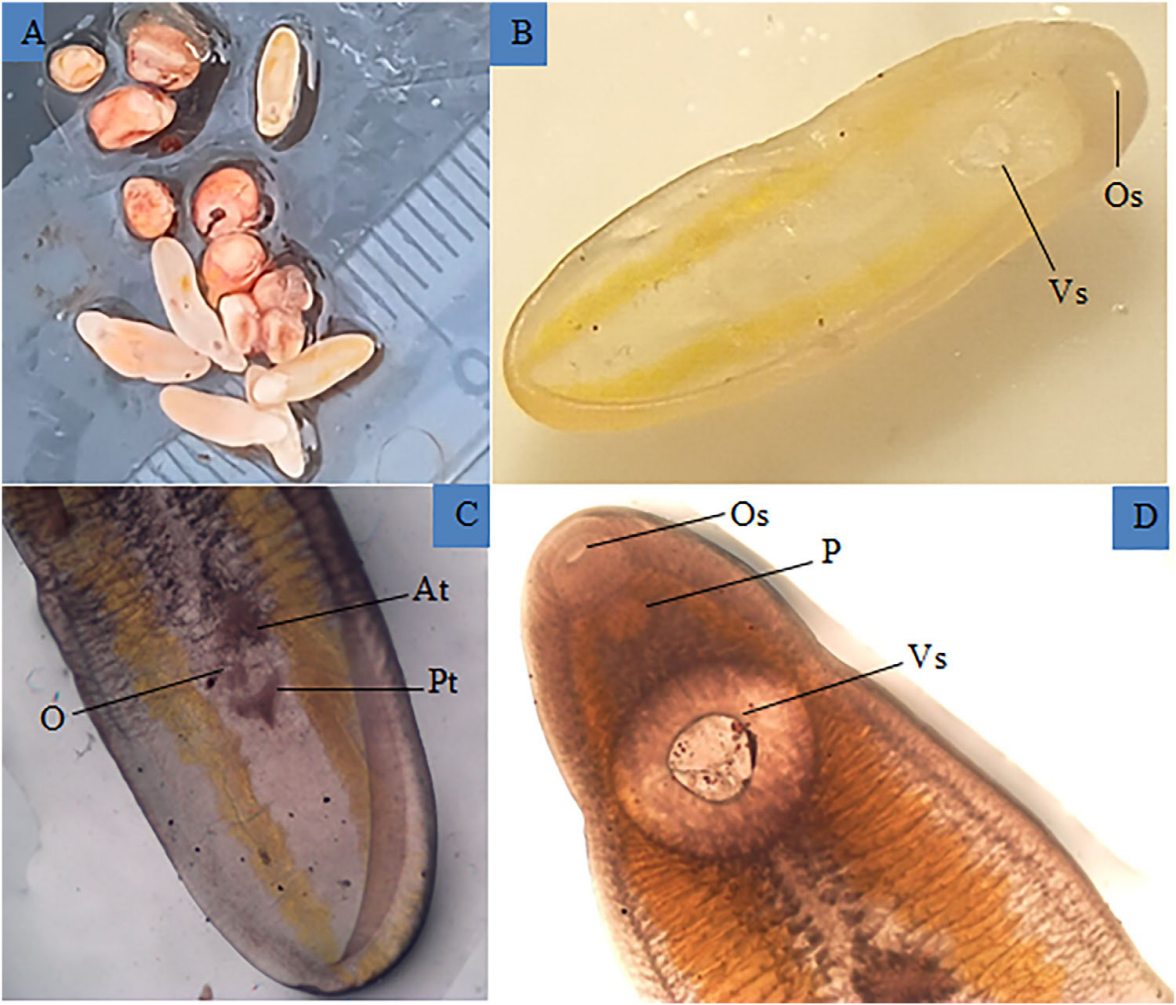
Macroscopic and microscopic morphology *of Clinostomum* metacercariae: (A) excysted metacercariae detected with the naked eye and (B) metacercariae identified under a stereomicroscope showing the oral sucker (Os) and ventral sucker (Vs) and (C and D) light microscopic appearance of *Clinostomum* metacercariae at 10× magnification, demonstrating anterior testis (At), posterior testis (Pt), ovaries (O), oral sucker (Os), pharynx (P) and ventral sucker (Vs).

### 3.2 Burden and Distribution of *Clinostomum* MC Infection

The distribution of EMC in the examined Nile tilapia was different between organs and study sites. Overall, the muscle revealed the highest proportion of EMC 83.3%, followed by gill 50% and liver 36.1% among the examined fish (Table 1). A higher proportion was observed in fish from Lake Tana, where EMC were detected in 94.4% of musculature, 66.6% of gills and 50% of liver samples, compared to 72.2%, 33.3% and 22.2%, respectively, in pond fish. In line with this, the total cyst burden was higher in Lake Tana 239 cysts than in the pond 57 cysts. Based on the proportional distribution of cysts, musculature revealed the largest proportion of the total cyst burden in both sites, 59.4% in Lake Tana and 56.1% in the pond, followed by gills 29.28% and 26.3% and liver 11.29% and 17.5%, respectively (Table 2). Usually, musculature was the major site of EMC infestation, followed by gills, whereas liver contribution remained comparatively lower across both study sites.

**TABLE 1:** Proportion of infected fish with cysts in each examined organs.

Infested organs	Lake *n* = 36 (%)	Pond *n* = 36 (%)	Total *n* = 72 (%)
Muscle	34 (94.4)	26 (72.2)	60 (83.3)
Gills	24 (66.6)	12 (33.3)	36 (50)
Liver	18 (50)	8 (22.2)	26 (36.1)

*Note*: Fish were collected using a purposive sampling technique to obtain representative specimens from the study areas. Therefore, the results of this study reflect only the examined fish and may not be generalized to the entire population.

**TABLE 2:** Distribution of cysts in target organs of Nile tilapia from the study area.

Site type	Total cysts	Muscle *n* (%)	Gills *n* (%)	Liver *n* (%)
Lake Tana	239	142 (59.4)	70 (29.28)	27 (11.29)
Pond	57	32 (56.1)	15 (26.3)	10 (17.5)

*Note*: Percentages indicate the proportion of cysts in each organ relative to the total cysts recorded per site.

### 3.3 Gross Pathological Findings

In the present study, the target organs of all sampled Nile tilapia (*O. niloticus*) were examined macroscopically. The investigation identified several gross lesions, mainly attributable to parasitic infection. Observed alterations included yellowish discolouration of EMC in gills, muscles and livers; excessive mucus deposition on the gills and body surface and hyperaemia of the gills and muscles, as well as pallor, necrosis and mottling of the gill tissues. Representative gross lesions induced by the parasite are presented in Figures 3, 4, 5 as photo macrographs.

**FIGURE 3:**
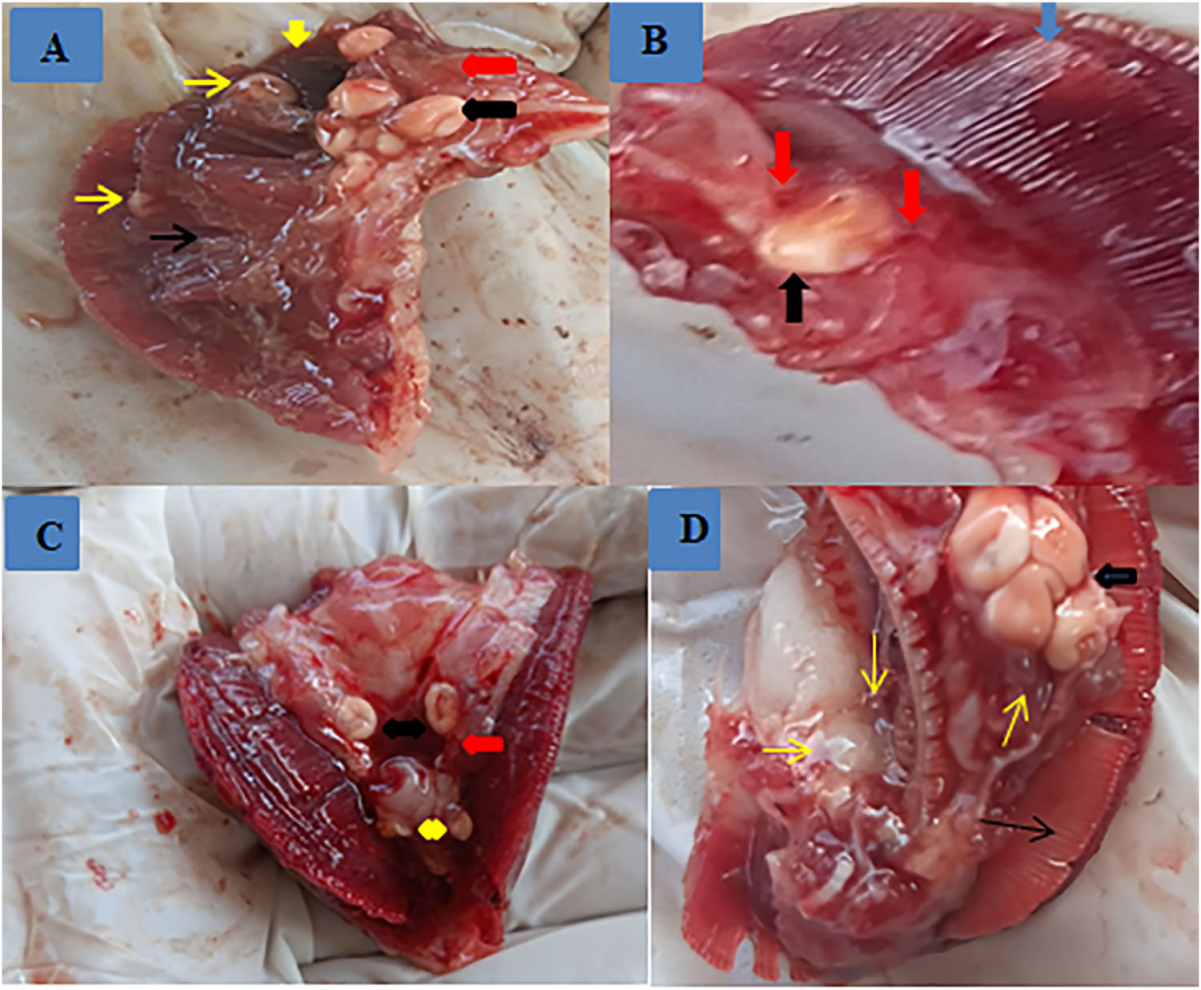
Gross pathological findings of fish gill: (A) Gill infected with encysted *Clinostomum* metacercariae located on the gill arch (thick black arrows) and filaments (thin yellow arrows), associated with localized inflammation (red arrow) and mucus secretions (thin black arrow); affected gill filaments exhibit necrosis and a mottled appearance (thick yellow arrow) from Lake Tana. (B) Creamy white to yellowish cysts observed on the gill arch (black arrow), accompanied by lightened and degenerated gill filaments (blue arrow) and marked hyperaemia (red arrow) surrounding the cysts from the pond. (C) Presence of cysts on the gill arch (black arrow) and chamber structure (yellow arrow), with evident surrounding hyperaemia (red arrow) indicative of inflammatory response in fish from the pond. (D) Presence of multiple cysts on the gill arch (thick black arrow) with evident mucus secretion (yellow arrow) and pallor of the gill filament (thin black arrow) from Lake Tana.

**FIGURE 4:**
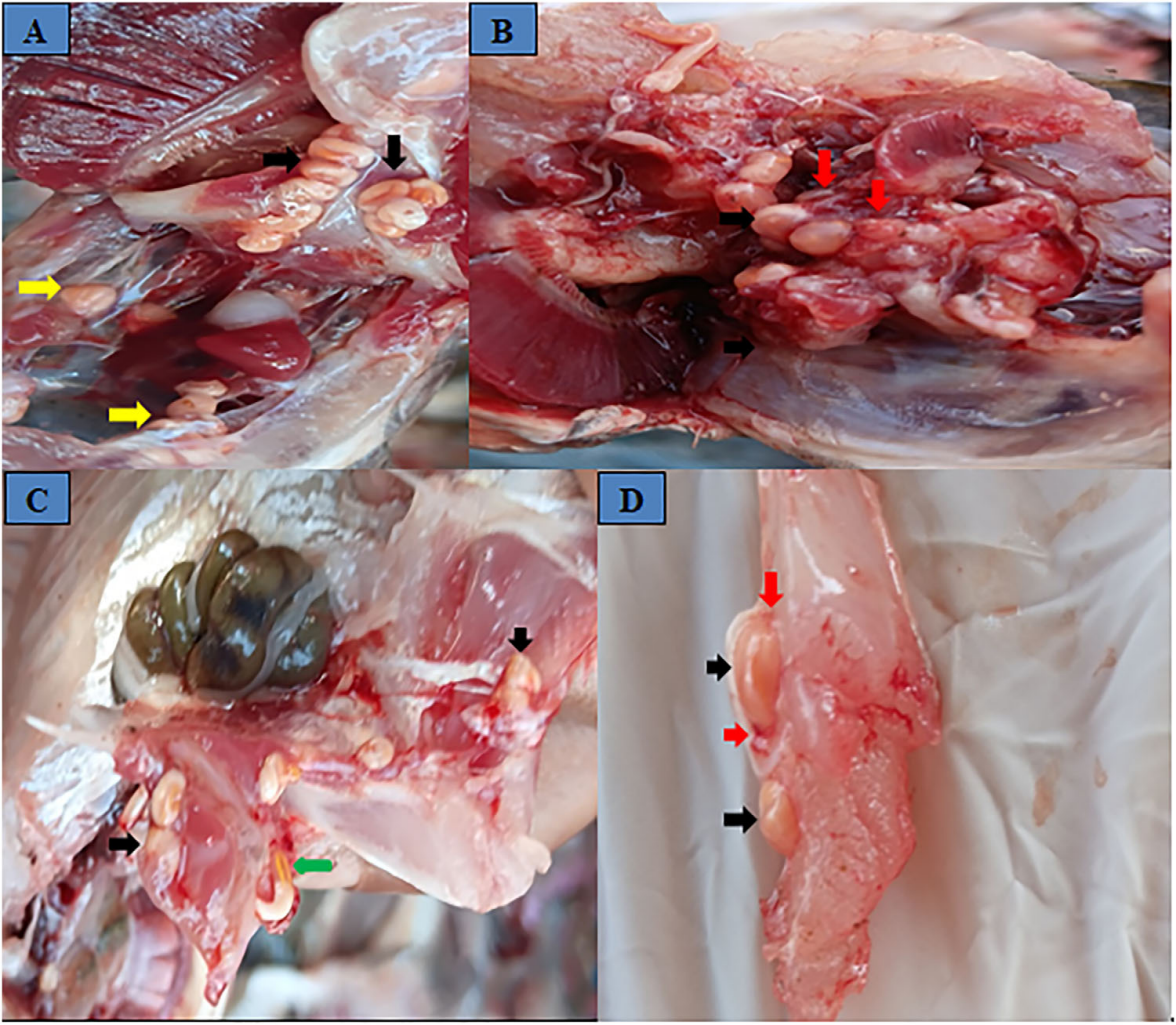
Gross pathological findings of fish musculature: (A) Distinct yellow to cream‐coloured cysts embedded within the muscle tissue (black arrow) and within the muscle fascia (yellow arrow) in the branchiostegal muscle region from Lake Tana. (B) Encysted metacercariae located within the buccal cavity tissue (black arrows), with surrounding hyperaemia (red arrow) indicative of localized inflammatory response from Lake Tana. (C) White to yellowish encysted metacercariae are observed within the musculature inside the body cavity (internal musculature) (black arrow), and the green arrow indicates live excysted metacercariae from the pond. (D) Encysted metacercariae are present in the musculature outside the body cavity (external musculature) (black arrow) and are associated with evident surrounding hyperaemia (red arrow), consistent with an inflammatory reaction from Lake Tana.

**FIGURE 5:**
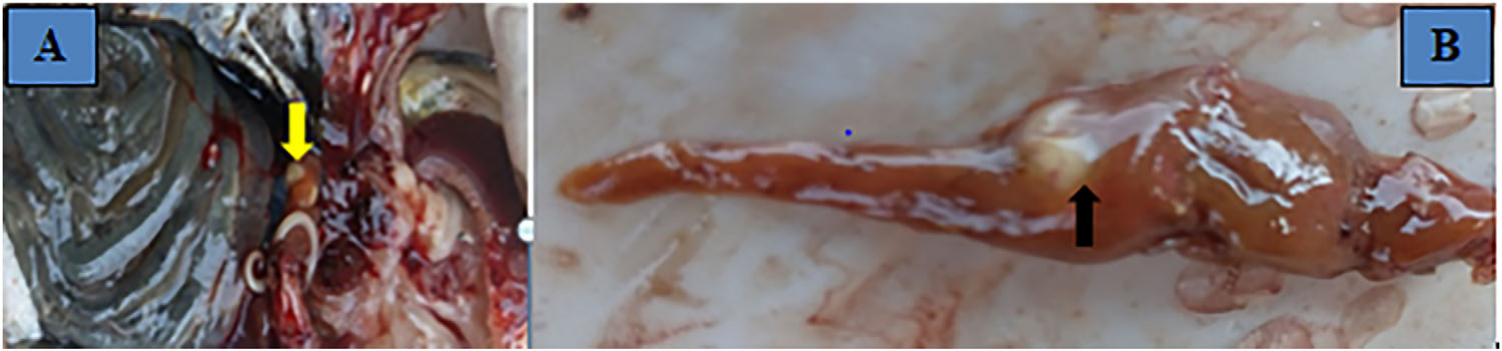
Gross pathological findings of the liver: (A) Encysted metacercariae present within the liver (yellow arrow); the liver is observed in situ from the pond. (B) Encysted metacercariae seen inside round yellowish capsules (black arrow) in the liver; the liver was examined ex situ from Lake Tana.

### 3.4 Gill Histopathological Findings and Percentage Frequency

In the current study, various histopathological alterations were observed in the gill tissues of sampled Nile tilapia. The detected lesions included haemorrhage, cellular infiltration, melanomacrophage centre (MMC), clubbing of secondary lamellae, vascular congestion, aneurysm, epithelial lifting and lamellar necrosis. The frequency of fish affected by each specific histopathological lesion in the gills was expressed as percentage frequency. Among the detected lesions, lamellar fusion (47.2%) and lamellar hyperplasia (44.4%) were the most frequent pathological changes observed in fish collected from the southern Gulf of Lake Tana compared with 25% and 22.2%, respectively, from pond fish. The total frequency of lamellar fusion and lamellar hyperplasia was 36.1% and 33.3%, respectively.

Haemorrhage was detected only in fish collected from the lake site and was absent in Nile tilapia fish from the pond site. Overall, the frequency of histopathological lesions was higher in fish sampled from the lake environment than in those collected from the pond. The percentage frequency of gill histopathological alterations associated with the study sites is provided in Table 3, and some of the representative microscopic lesions and their descriptions are illustrated in Figure 6.

**TABLE 3:** Percentage frequency of gill histological lesions in infected fish.

Lesion	Lake *n* = 36 (%)	Pond *n* = 36 (%)	Total *n* = 72 (%)
Haemorrhage	2 (5.55)	—	2 (2.77)
Lamellar hyperplasia	16 (44.4)	8 (22.2)	24 (33.3)
Cellular infiltration	5 (13.88)	2 (5.55)	7 (9.7)
Melanomacrophage	3 (8.3)	1 (2.77)	4 (5.55)
Lamellar fusions	17 (47.2)	9 (25)	26 (36.1)
Clubbing of secondary lamellae	4 (11.1)	2 (5.55)	6 (8.3)
Vascular congestion	6 (16.66)	2 (5.55)	8 (11.1)
Aneurysm	5 (13.88)	2 (5.55)	7 (9.7)
Epithelial lifting and lamellar necrosis	4 (11.1)	1 (2.77)	5 (6.9)

*Note*: The ’—‘ indicates the absence of haemorrhage in fish sampled from the pond site.

**FIGURE 6:**
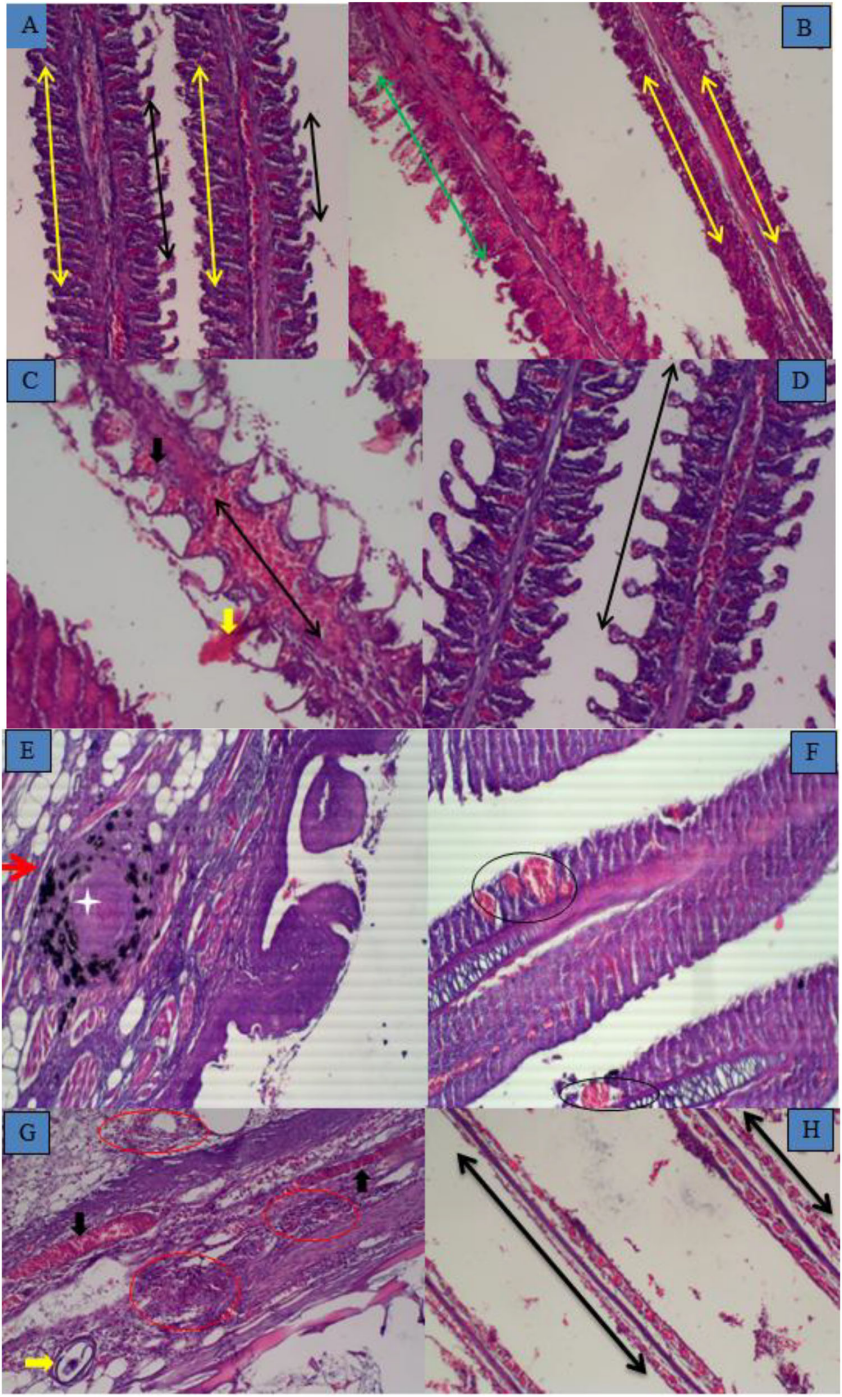
Microscopic lesions of gill: (A) apparently healthy gill lamellae (double black arrow) and moderate fusion of secondary lamellae with epithelial hyperplasia (double yellow arrow) from the pond; (B) epithelial lifting and secondary lamellar necrosis (double green arrow), with severe secondary lamellar fusion and hyperplasia (double yellow arrow) from Lake Tana; (C) vascular congestion of capillaries in both the primary lamellae (double black arrow) and secondary lamellae (thick black arrow), with haemorrhage (yellow arrow) from Lake Tana; (D) clubbing of secondary lamellae (double black arrow) from Lake Tana; (E) granulomatous lesion likely associated with metacercarial encystment (star) in the gill arch, with MMC (red arrow) from Lake Tana; (F) aneurysm (circle) with severe lamellar fusion from the pond; (G) vascular congestion (black arrow), cellular infiltration (circle) and metacercarial encystment in the gill arch (yellow arrow) from Lake Tana and (H) severe lamellar necrosis and epithelial lifting (double black arrow) from Lake Tana. Magnification, 10×; hematoxylin and eosin (H&E) stain.

### 3.5 Muscle Histopathological Findings and Percentage Frequency

Different histopathological lesions were detected in the muscle tissues of Nile tilapia fish collected from both Lake Tana and adjacent ponds. The detected lesions included haemorrhage, cellular infiltration, muscle fibre degeneration and disruption and fibrous connective tissue proliferation. The lesions were recorded and expressed as percentage frequency on the basis of the number of affected fish.

Among the detected lesions, muscle fibre degeneration and disruption (83.3%) and cellular infiltration (41.6%) were the most frequent pathological changes observed in fish collected from the southern Gulf of Lake Tana, compared with 50% and 25%, respectively, from pond fish. The total percentage frequency of muscle fibre degeneration and disruption and cellular infiltration was 66.6% and 33.3%, respectively. Haemorrhage was the least observed lesion, detected in 8.3% of fish from the lake and 2.77% from the pond, with a total frequency of 5.55%.

Generally, all recorded muscle histopathological lesions revealed higher percentage frequency in Nile tilapia fish sampled from the Lake Tana compared to the pond site. The detailed percentage frequency of muscle histological alterations and their associated with study sites are provided in Table 4, and some of the histological lesion descriptions are presented in Figure 7.

**TABLE 4:** Percentage frequency of muscle histological lesions in infected fish.

Lesion	Lake *n* = 36 (%)	Pond *n* = 36 (%)	Total *n* = 72 (%)
Haemorrhage	3 (8.3)	1 (2.77)	4 (5.55)
Cellular infiltration	15 (41.6)	9 (25)	24 (33.3)
Muscle fibre degeneration and disruption	30 (83.3)	18 (50)	48 (66.66)
Fibrous connective tissue	12 (33.3)	8 (22.2)	20 (27.77)

**FIGURE 7:**
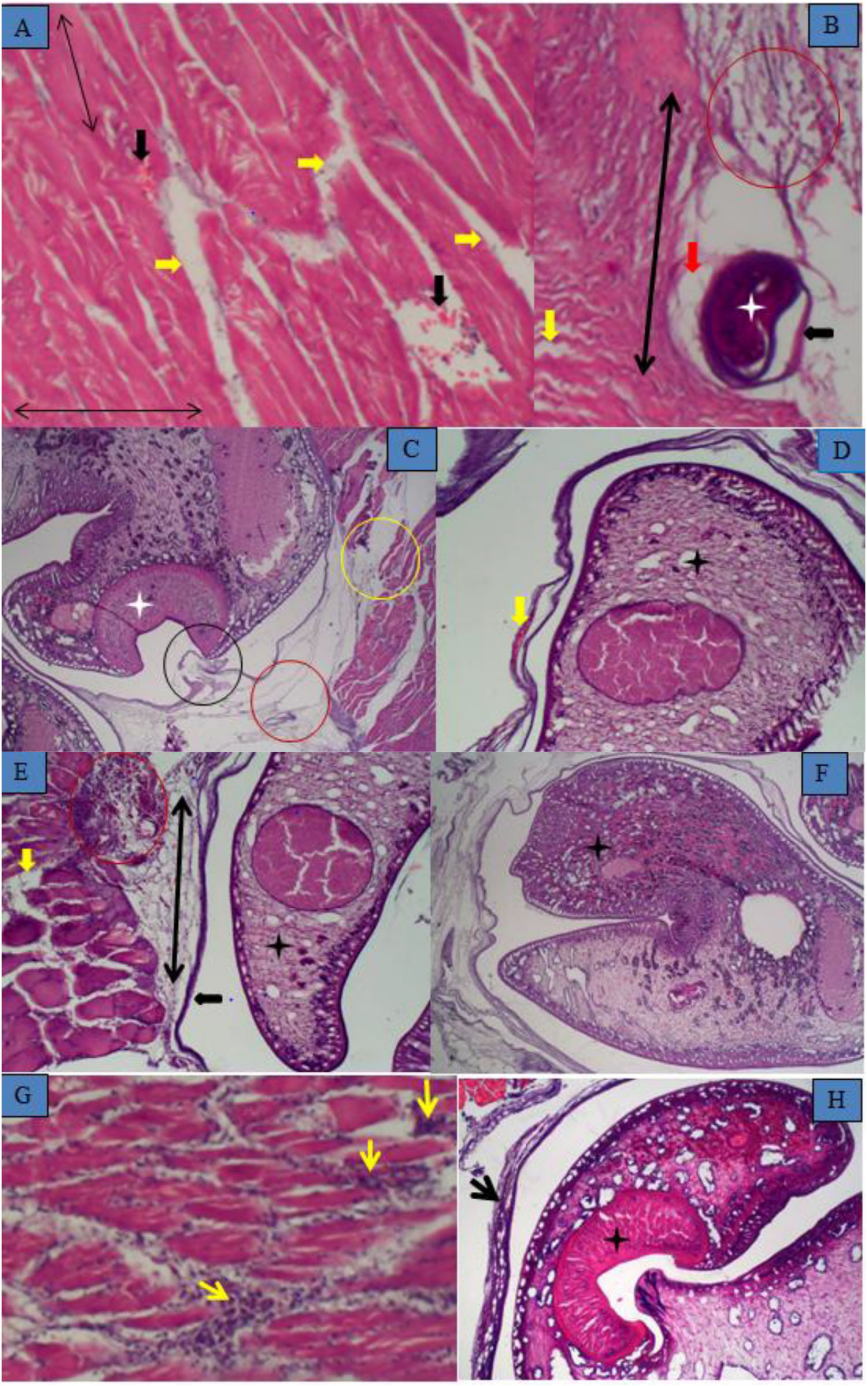
Microscopic Lesion of musculature: (A) apparently healthy muscle (double black arrow) with haemorrhage (black arrow) and muscle fibre splitting (yellow arrow) from Lake Tana; (B) encysted metacercariae in the muscle (star), with fibre disruption around the encysted metacercariae (red arrow), loss of muscular organization (double black arrow) and cellular infiltration (circle) from Lake Tana; (C) cross‐section of the *Clinostomum* metacercaria oral sucker (white star), engulfed tissue by the *Clinostomum* metacercariae sucker (black circle), with severe muscle fibre disruption (yellow circle) and necrosis (red circle) around the cyst from Lake Tana; (D) encysted metacercariae observed within the muscle tissue (star), surrounded by fibrous connective tissue capsules with capsular haemorrhage (yellow arrow) from the pond; (E) encapsulated metacercariae within the muscle tissue (star), marked muscle degeneration (double black arrow), muscle fibre splitting (yellow arrow) and abundant leukocyte infiltration (red circle) around the cyst from Lake Tana; (F) encapsulated metacercariae within the muscle fasciae (star) from pond; (G) heavy cellular infiltration in the infected muscle tissue (yellow arrow) from Lake Tana and (H) cross‐section of the encysted metacercariae with ventral sucker (star) and cellular infiltration in the fibrous capsule (black arrow) from Lake Tana. Magnification, 10×; hematoxylin and eosin (H&E) stain.

### 3.6 Liver Histopathological Findings and Percentage Frequency

Different histopathological lesions were observed in the liver tissues collected from both Lake Tana and adjacent ponds. The detected lesions included haemorrhage, hepatocyte necrosis, hepatocyte vacuolation, cellular infiltration, sinusoidal dilation and congestion, central vein dilation and congestion, biliary hyperplasia, MMC and fibrous connective tissue proliferation.

Among the detected lesions, sinusoidal dilation and congestion and hepatocyte vacuolation were the most alterations, with percentage frequencies of 19.4% and 16.66%, respectively, from Lake Tana, compared with 8.3% and 5.55%, respectively, from pond fish. The total percentage frequency of sinusoidal dilation and congestion and hepatocyte vacuolation was 13.88% and 11.1%, respectively. Biliary hyperplasia was the least observed lesion from both study sites.

Generally, liver lesions were more frequently observed in fish collected from the Lake Tana than in those from ponds. Percentage frequencies of liver histopathological lesions associated with the study sites are present in Table 5, and some representative liver lesions and their descriptions are present in Figure 8.

**TABLE 5:** Percentage frequency of liver histological lesions in infected fish.

Lesion	Lake *n* = 36 (%)	Pond *n* = 36 (%)	Total *n* = 72 (%)
Haemorrhage	5 (13.88)	2 (5.55)	7 (9.7)
Hepatocyte necrosis	4 (11.1)	3 (8.3)	7 (9.7)
Hepatocyte vacuolation	6 (16.66)	2 (5.55)	8 (11.1)
Cellular infiltration	3 (8.3)	1 (2.77)	4 (5.55)
Sinusoidal dilation and congestion	7 (19.4)	3 (8.3)	10 (13.88)
Central vein dilation and congestion	4 (11.1)	3 (8.3)	7 (9.7)
Biliary hyperplasia	2 (5.55)	1 (2.77)	3 (4.16)
Melanomacrophage	4 (11.1)	2 (5.55)	6 (8.3)
Fibrous connective tissue	3 (8.3)	2 (5.55)	5 (6.9)

**FIGURE 8:**
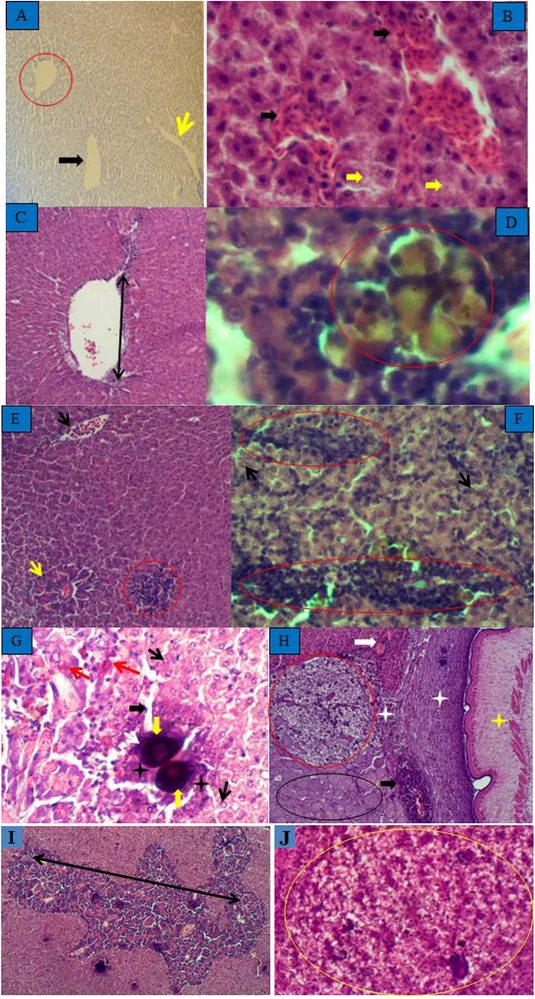
Microscopic lesion of liver: (A) relatively normal hepatocytes, central vein (circle), hepatic vein (black arrow) and sinusoid (yellow arrow) from pond (10×); (B) central vein congestion with inflammatory cell infiltration (black arrow) and hepatocellular necrosis (yellow arrow) from Lake Tana (40×); (C) dilated central vein with minimal congestion (double black arrow) from pond fish (10×); (D) melanomacrophage aggregation and inflammatory cellular infiltration (circle) from Lake Tana (40×); (E) sinusoidal dilation and congestion (black arrow) with cellular infiltration (circle) and mild biliary hyperplasia (yellow arrow) from pond (10×); (F) marked cellular infiltration (circle) and hepatocellular vacuolation (black arrow) from Lake Tana (40×); (G) two encysted metacercaria (yellow arrow) within the liver parenchyma surrounded by a fibrous capsule (white arrow) and cellular infiltration (star), with sinusoidal dilation (thick black arrow), congestion (red arrow) and hepatocyte vacuolation (thin black arrow) from Lake Tana (40×); (H) cross‐sectional view of encysted metacercaria (yellow star) enclosed by fibrous connective tissue capsule with cellular infiltration (white star), granulomatous lesion (red circle) with hepatocyte degeneration (black circle), mild biliary hyperplasia (black arrow) and haemorrhage (white arrow) from Lake Tana (10×); (I) the liver exhibited marked biliary hyperplasia (double black arrow) from Lake Tana (40×) and (J) hepatocellular necrosis (yellow circle) from Lake Tana (40×). Hematoxylin and eosin (H&E) stain.

### 3.7 Mean Values of Organ Histopathological Index Severity

The present study assessed the mean severity scores of histopathological lesion indices in the target organs. The mean severity scores of each organ in relation to the study sites were analysed using an independent sample *t*‐test.

Collected fish from Lake Tana had higher mean organ severity indices than those obtained from ponds. The mean gill severity index was 2.38 in fish from Lake Tana and 0.55 in fish from the pond, with the total mean value of 1.47. Similarly, the mean muscle severity index was higher in fish from Lake Tana 3.13 than in fish from pond 1.13, with a total mean value of 2.13. The mean liver severity index was also higher in fish collected from Lake Tana 1.4 compared to fish from pond 0.58, with an overall mean value of 1.01.

Among the examined organs, muscle tissue was the most affected organ, showing the highest total mean severity index of 2.13, followed by the gills 1.47, whereas the liver was the least affected organ with a mean severity index value of 1.01. In addition, the fish severity index was higher in Lake Tana 6.97 compared to pond 2.27, with the total mean value of fish index 4.6. Generally, the analysis revealed statistically significant differences between the two study sites for all examined organs and the total fish index (Table 6).

**TABLE 6:** Mean, SD of organ severity index level and its significance state.

Organ (severity)	Lake *n* = 36 mean(±SD)	Pond *n* = 36 mean(±SD)	Total *n* = 72 mean(±SD)	*T* value	*p* value
Gill severity	2.38 (2.18)	0.55 (0.8)	1.47 (1.87)	4.72	<0.001
Muscle severity	3.13 (1.67)	1.13 (1.07)	2.13 (1.7)	6.03	<0.001
Liver severity	1.4 (1.7)	0.58 (1.2)	1.01 (1.55)	2.43	0.018
Fish index	6.97 (3.01)	2.27 (1.23)	4.6 (3.29)	8.63	<0.001

### 3.8 Correlation of Parasite Burden With Organ Lesion Indices

The present study assessed the relationship between parasite burden and the histopathological lesion indices of the target organs. Pearson's correlation test was used to determine the correlation between parasite burden and organ lesion indices in the two study sites. Collected fish from both Lake Tana and pond, gill parasite burden revealed strong positive correlation with the gill lesion index. Similarly, muscle parasite burden also revealed a strong positive correlation with the muscle lesion index. The liver lesion index exhibited a very strong positive correlation with liver parasite burden. Moreover, the total fish index had a strong positive correlation with the total parasite burden in Lake Tana and moderate correlation in the pond. Generally, the analysis revealed statistically significant positive correlations between parasite burden and the lesion indices of all examined organs, as well as the total fish index, in both study sites. The detailed correlation coefficients and significance values are presented in Table 7.

**TABLE 7:** Correlation between parasite burden and organ histopathological indices.

Parameter	Lake (*r*)	*p* value	Pond (*r*)	*p* value	Interpretation
Gill index vs. gill parasite burden	0.7	<0.001	0.85	<0.001	Strong positive
Muscle index vs. muscle parasite burden	0.8	<0.001	0.79	<0.001	Strong positive
Liver index vs. liver parasite burden	0.89	<0.001	0.9	<0.001	Very strong positive
Fish index vs. total parasite burden	0.78	<0.001	0.6	<0.001	Strong positive moderate in pond

*Note*: Correlation coefficients (*r*) were calculated separately for each study site using Pearson correlation. *p* values <0.05 were considered statistically significant.

## 4 Discussion

The fishery sector plays a crucial role in food security through the supplementation of protein source food for the world population, particularly in developing countries like Ethiopia. However, one of the major problems of the fishery sector is trematode parasite infection, which affects aquaculture production worldwide, in addition to posing potential zoonotic risks to human health (Rediet et al. 2022; Abd‐ELrahman et al. 2023). Among the trematode parasite groups, the *Clinostomum* metacercaria is the most important endoparasite responsible for great economic losses for both wild and cultured fishes (Yassen et al. 2023).

The present study provided baseline information on the *Clinostomum* MC in *O. niloticus* from Lake Tana and nearby ponds. Morphological identification of the MC was successfully carried out using naked eye, stereomicroscope and light microscope. The detected EMC were characterized by yellowish‐white to orange pea‐like cysts, stout, tongue‐shaped bodies with a flattened, non‐segmented structure, smooth lateral margins and a complete absence of spines. Additionally, internal structures of MC, including the anterior and posterior testes, ovaries, oral sucker, pharynx and ventral sucker, were detected and used to confirm the genus‐level of the *Clinostomum* MC. This method agrees with previous studies that used similar morphological features for MC identification (Zimik et al. 2019; Yassen et al. 2023; Mahdy et al. 2024; Aydogdu et al. 2025).

Although genus‐level identification was successfully confirmed via traditional light and stereomicroscopy, recent investigations highlight that aquatic ecosystems in this region harbour more than two distinct types of *Clinostomid* parasites; specifically, the zoonotic pathogen *Clinostomum complanatum* alongside non‐zoonotic counterparts (Mahdy et al. 2024; Salem et al. 2025). As demonstrated by Mahdy et al., distinguishing between these species requires precise morpho‐molecular characterization. Consequently, researchers studying these ecosystems must exercise caution to accurately identify the specific species under investigation and determine whether their samples comprise a single uniform species or multiple co‐infecting species.

In the present study, the distribution of recovered EMC was higher in muscle 83.3%, followed by gill 50% and liver 36.1% among the examined fish. Moreover, the highest frequency rate of EMC was observed in fish from Lake Tana, 94.4% of musculature, 66.6% of gills and 50% of liver samples, compared to 72.2%, 33.3% and 22.2%, respectively, from pond fish. The total cyst burden was higher in Lake Tana 239 cysts than in the pond 57 cysts. Based on the proportional distribution of MC, musculature was the highest proportion from the total cyst burden in both study sites, 59.4% in Lake Tana and 56.1% from pond fish, followed by gills 29.28% and 26.3% and liver 11.29% and 17.5%, respectively. This finding agrees with previous studies showing that the muscles are the most commonly affected organs by EMC, followed by the gill (Eissa et al. 2011; Salem et al. 2025). The eggs of *Clinostomum* species hatch into ciliated miracidia in water, which then invade freshwater snails, their first intermediate host (Tavares‐Dias et al. 2023). Within the snail, the parasite undergoes successive developmental stages, including sporocyst, redia and cercaria (Choudhary et al. 2022). Researchers suggested that cercariae of *Clinostomum* spp. enter with water through the oral cavity, gill and operculum and can easily attach to soft tissues compared to the scaly body surface. These parasites may feed on the mucous, blood and tissue from the host, so they are commonly found in the branchial cavity and beneath the branchiostegal muscle, and leading to pathological changes and alterations in fish behaviour, which can result in significant economic losses in aquaculture operations (Yassen et al. 2023; Mahdy et al. 2024). Generally, this study revealed a significant difference in infection rates between the lake and the pond fish. These variations may be associated with differences in habitat and environmental conditions, including water temperature, the abundance of first intermediate hosts and the presence of piscivorous birds, which play a major role in completing the life cycle of *Clinostomum* parasites (Zimik et al. 2019; Montes et al. 2020; Mahdy et al. 2024).

Gross and histopathological investigations are crucial for assessing how *Clinostomum* MC affect organ function and overall fish health. Therefore, the present study was conducted to examine the pathological lesions of *Clinostomum* MC in Nile tilapia fish species inhabiting both Lake Tana and the adjacent pond. This finding revealed that some gross pathological lesions, including yellowish discolouration of EMC in gill, muscles and livers, excessive mucus deposition on gills and body surface, hyperaemia in gill and muscle, pallor, necrosis and mottled appearance of the gill tissues. These findings are in agreement with previous studies that reported similar pathological changes in fish infected with MC (Shareef and Abidi 2012; Montes et al. 2020; Yassen et al. 2023; Mahdy et al. 2024).

In this finding, histopathological assessment was performed using both qualitative and quantitative methods. The qualitative assessment involved the histopathological lesion detection, identification and detailed description of observed organs. These observed lesions were then converted into quantitative data using the lesion scoring system previously described by Bernet et al. (1999).

The current study revealed numerous histopathological lesions in the gill tissues of *O. niloticus* infected with EMC. The observed lesions include lamellar hyperplasia, lamellar fusion, haemorrhage, cellular infiltration, MMC, clubbing of secondary lamellae, vascular congestion, aneurysm, epithelial lifting and lamellar necrosis. These lesions were nearly similar to those described by Clinton et al. (2024), Ramadan et al. (2024) and Mohammed and Ramadan (2025). Among the detected lesions, lamellar fusion and lamellar hyperplasia were the most frequent lesions in both study sites. These lesions are considered common defensive and adaptive responses of gill tissues against parasitic irritation and tissue damage. Hyperplasia of epithelial cells may subsequently result in fusion of adjacent secondary lamellae, thereby reducing the respiratory surface area and impairing gaseous exchange. Similar findings have been reported by Blazer and Gratzek (1985) and Hossain et al. (2007). The occurrence of haemorrhage, vascular congestion and aneurysm in the present study may indicate circulatory disturbances and vascular damage in the gill tissues. These changes may result from mechanical pressure exerted by EMC and inflammatory reactions within the branchial tissues. These lesions were nearly similar to those observed by Blazer and Gratzek (1985) and Amlie et al. (2025). Epithelial lifting and lamellar necrosis were detected in the present work, suggesting severe tissue degeneration of the fish gill structure. Similar pathological lesions have been reported in fish infected with gill parasites and MC (Pramanik and Biswas 2024; Ramadan et al. 2024). Inflammatory reactions characterized by cellular infiltration and melanomacrophage aggregation were also detected in infected gill tissues. These findings likely reflect activation of the host immune response against parasitic irritation and tissue injury. MMC is commonly associated with phagocytosis, inflammatory responses and removal of damaged cellular components during chronic infection and stress conditions in fish tissues. Similar inflammatory responses have been reported previously by Silva et al. (2016) and Salem et al. (2025).

The present study revealed several histopathological lesions in the muscle tissues of Nile tilapia fish collected from both Lake Tana and adjacent pond environments. Muscle fibre degeneration and disruption was the most frequent lesion in both study sites. This finding agrees with the reports of Shareef and Abidi (2012), Yassen et al. (2023), Mahdy et al. (2024) and Aydogdu et al. (2025), who documented severe tissue degeneration and destruction associated with metacercarial infections. Degeneration and disruption of muscle fibres are commonly associated with the migratory action and encystment of MC within muscle tissues, leading to mechanical damage and necrosis of surrounding fibres (Echi et al. 2009). Marked inflammatory cell infiltration observed in the muscle tissue may reveal the defensive response of the host against infesting parasites or tissue damage caused by the EMC. This finding is similar to the observations of Zimik et al. (2019); they described inflammatory cell infiltration surrounding EMC. In this study, fibrous connective tissue proliferation was detected, which may reflect a chronic tissue repair response caused by prolonged parasitic irritation and muscle damage. This finding agrees with previous studies of Montes et al. (2020), Mahdy et al. (2024) and Salem et al. (2025). Haemorrhage was the least frequently observed lesion in the current study. However, its presence may suggest damage to blood vessels associated with *Clinostomum* parasite migration. This observation is related to the findings of Silva et al. (2016).

The liver is the vital organ used for detoxification and biotransformation processes and, due to its high metabolic activity, rich blood supply and direct connection to the gastrointestinal circulation, is mostly exposed to pathological changes (Olsvik et al. 2007). In this study, several histological lesions were detected in the liver, including haemorrhage, hepatocyte necrosis, hepatocyte vacuolation, inflammatory cell infiltration, sinusoidal dilation and congestion, central vein dilation and congestion, biliary hyperplasia, melanomacrophage aggregation and fibrous connective tissue proliferation. This finding is supported by the previous works (Islam et al. 2023; Yassen et al. 2023; Guelmamene et al. 2025). Among the identified lesions, sinusoidal dilation and congestion and hepatocyte vacuolation were the most common changes in the study site. These changes suggest disorders of normal blood circulation within the liver and damage to hepatocellular function, which may be due to prolonged exposure to parasitic infection and is associated with physiological stress (Olsvik et al. 2007; Montes et al. 2020; Guelmamene et al. 2025). Hepatocyte necrosis and haemorrhage were also detected, indicating localized tissue injury and likely rupture of blood vessels. Typically related to parasite migration and the release of toxins that compromise cellular integrity, central vein dilation and congestion further support the occurrence of circulatory disturbances within the hepatic tissue, which may affect the normal liver function and metabolic processes (Tansatit et al. 2014; Islam et al. 2023; Yassen et al. 2023). Inflammatory cell infiltration was detected in the fish liver, indicating an active immune response against the EMC invasion. In addition, melanomacrophage aggregation suggests chronic infection of the EMC and association with clearing of cellular debris and pathogens. These lesions are similar to the work of Khalil et al. (2014), Mahdy et al. (2023) and Guelmamene et al. (2025). In this study biliary hyperplasia was also observed, indicating chronic irritation of the bile ducts due to the prolonged parasite exposure. Similarly, fibrous connective tissue proliferation was observed, indicating chronic tissue repair response caused by prolonged parasitic irritation and liver damage (Eissa et al. 2011; Zimik et al. 2019; Mahdy et al. 2023). Generally, the liver exhibited more pathological lesions in Lake Tana compared to those from the adjacent pond, suggesting that environmental conditions and parasite exposure levels in the lake may have contributed to greater hepatic tissue injury (Stumbo et al. 2012; Berg et al. 2013).

According to the lesion scoring system, the severity of histopathological lesions in each gill, muscle and liver was detected. The present study showed a mean value of muscle 2.13 and followed by gill 1.47 and the smallest mean values in the liver 1.01. These findings indicate that muscles are highly affected organs as compared to other organs, and the result is supported by Roberts (2012). Similarly, the mean value of fish index was largely higher in fish from the southern Gulf of Lake Tana (6.97) compared with the pond site (2.27), this indicates fish from the southern Gulf of Lake Tana were exposed to a higher parasitic burden, uncontrolled environmental conditions and higher parasite transmission dynamics, and other factors like feed quality or quantity might also further expose the fish to many stressors (Chai et al. 2005), and fish from adjacent ponds have good management interventions like proper feeding, water refreshments and fencing; those factors may improve the adaptive nature of the Nile tilapia and result in lower severity of histological change (Getnet et al. 2024).

The present study indicated strong positive correlation between parasite burden and histopathological lesion severity indices in all studied organs of fish from both Lake Tana and adjacent pond. This result indicates that as the number of EMC increased, the severity of histopathological lesions also increased (Bonett et al. 2011).

A strong positive correlation was observed between gill parasite burden and gill histopathological index in both Lake Tana and adjacent pond. This indicates that the higher numbers of EMC in the gill tissues are associated with more histological lesion severity (Salem et al. 2025). Similarly, muscle parasite burden revealed a strong positive correlation with muscle histopathological index in fish from Lake Tana and ponds. This indicates that the highest EMC infection is associated with the severe muscular damage (Shareef and Abidi 2012).

The liver exhibited the strongest correlation between the EMC burden and the histological lesion severity in both Lake Tana and pond fish. This finding indicates that increased the infection of EMC may severely affect liver tissue (Eissa et al. 2011). In addition, the fish index revealed a strong positive correlation between the total parasite burden in Lake Tana and moderate levels in pond fish. This shows fish harbouring larger numbers of parasites often faced more significant tissue damage and lower overall health state (Mahdy et al. 2023; Yassen et al. 2023). Generally, the present results clearly reveal that increasing parasite burden is strongly associated with severe histological changes in *O. niloticus*.

### 4.1 Limitations of the Study and Future Directions

Although this study provided valuable insights, certain limitations should be considered. The cross‐sectional study design offers only a snapshot in time and therefore may not fully represent seasonal variations in parasite occurrence. In addition, tissue sample transportation from Lake Tana to the pathology laboratory may also have contributed to partial sample degradation, which may affect the quality and clarity of histopathological image observations. The use of cervical dislocation may cause localized haemorrhage, potentially confusing the interpretation of parasite‐associated lesions. These were considered during the pathological lesion examination. Therefore, future studies should use other euthanasia methods to differentiate the procedure‐related and parasite‐induced lesions. Parasite‐negative control groups of fish, concurrent infections and water pollution were not studied. Therefore, some of the lesions may not have been caused only by *Clinostomum* MC and may have influenced the lesion interpretation results. Furthermore, identification of *Clinostomum* metacercariea depends on morphological features because molecular diagnostic techniques were not available; therefore, we call for molecular screening to confirm the species level of the parasite. The observed correlations are interpreted primarily as reflecting local cyst‐associated tissue damage rather than an independent systemic dose‐dependent pathological effect. Financial and laboratory resource limitations also limited the extent of advanced histopathological analysis and detailed photographic documentation.

## 5 Conclusion and Recommendations

Based on the literature, this finding is the first pathological study of *Clinostomum* MC infection in *O. niloticus* from Lake Tana and adjacent ponds. The results indicate *Clinostomum* MC are very important helminth parasites of *O. niloticus*, with a higher total parasite burden in Lake Tana compared to the adjacent pond. Among the examined organs, the muscle tissue exhibited the highest parasite burden compared to the gill and liver. Several gross and histopathological lesions associated with the EMC were identified, and both of histological lesions and mean severity were greater in fish from Lake Tana. Moreover, the mean total histopathological fish index result was significantly higher at the southern gulf of Lake Tana as compared to the adjacent pond. In addition, a significant positive correlation was observed between parasite burden and lesion severity scores in both study areas, indicating that increased parasite burden was associated with more severe tissue damage. Further detailed studies involving several sampling sites within the lake should be conducted to get more comprehensive evidence regarding the pathological effects of *Clinostomum* MC infection. Strengthening aquaculture farm biosecurity is essential to limit the spread of *Clinostomum* MC disease. Snails should be effectively controlled using convenient methods to decrease the spread of *Clinostomum* MC. Responsible bodies should regularly conduct surveillance to protect both the lake and the pond water from *Clinostomum* MC infection. The community should be encouraged to consume properly cooked fish to reduce the risk of parasite transmission. Finally, future studies should consider seasonal variations and molecular techniques for the accurate identification and characterization of specific parasite species.

## Author Contributions

**Gebyaw Menge Getnet**: conceptualization, writing – original draft, methodology, writing – review and editing, software, formal analysis, data curation, resources, visualization, validation. **Mersha Chanie Kebede**: supervision, methodology, writing – review and editing, validation, visualization. **Assaye Desta Amare**: supervision, writing – review and editing, validation, visualization. **Ayenalem Shibabaw Atenaf**: supervision, validation, visualization. **Assaye Wollelie Fentie**: data curation, validation, visualization. **Hailu Mazengia**: supervision, methodology, writing – review and editing, validation, visualization.

## Funding

The authors have nothing to report.

## Ethics Statement

Ethical approval for animal sampling was obtained from the University of Gondar Research Ethics committee (Approval No. CVMAS‐012/2025). All procedures followed institutional and national guidelines for animal welfare.

## Consent

The authors have nothing to report.

## Conflicts of Interest

The authors declare no conflicts of interest.

## Data Availability

The datasets used and/or analysed during the current study are available from the corresponding author on reasonable request.
